# Mean arterial pressure during cardiopulmonary bypass: A modifiable risk factor for acute kidney injury in cardiac surgery patients?

**DOI:** 10.1186/s13054-024-04862-x

**Published:** 2024-03-12

**Authors:** Nikolaus Schreiber, Simon Orlob, Stephanie Fida, Christoph Klivinyi, Alexander H. Kirsch, Michael Kolland, Michael Schörghuber

**Affiliations:** 1 Division of Anaesthesiology and Intensive Care Medicine 2, Department of Anaesthesiology and Intensive Care Medicine, Medical University of Graz, Auenbruggerplatz 5, 8036 Graz, Austria; 2 Division of Nephrology, Department of Internal Medicine, Medical University of Graz, Graz, Austria

Dear Editor,

Acute kidney injury (AKI) frequently occurs as a complication in patients undergoing cardiac surgery, with reported incidence rates ranging from 20 to 40% [1]. The development of cardiac surgery-associated AKI (CSA-AKI) is linked to adverse outcomes such as increased short- and long-term mortality, as well as prolonged hospital stays [1].

Despite its significance, the underlying mechanisms of CSA-AKI remain inadequately understood and research concerning optimization of perioperative management to mitigate CSA-AKI is needed [2].

While a link between mean arterial pressure (MAP) under 65 mmHg and AKI has been established in noncardiac surgery patients [3], there is still controversy regarding this association in cardiac surgery patients, particularly during cardiopulmonary bypass (CPB), a critical period with nonpulsatile albeit constant flow, altered hemodynamics, decreased oxygen delivery and oxidative stress, each of which potentially contribute to CSA-AKI [2].

We hypothesized that an increase in time-weighted average (TWA) MAP under 65 mmHg during CPB is associated with the development of CSA-AKI.

To generate reproducible and transparent results, we analyzed the publicly available INSPIRE-research-dataset, which provides high-resolution multi-parameter data of patients who underwent anesthesia for surgery at an academic institution in South Korea between 2011 and 2020 [4].

All patients who underwent CPB during cardiac surgery with availability of invasive MAP data with at least five-minute resolution were included.

As primary outcome we used the occurrence of AKI according to Kidney Disease: Improving Global Outcomes (KDIGO) AKI-creatinine criteria (all stages) within 7 days of surgery [5]. As secondary outcome we evaluated the incidence of continuous renal replacement therapy (CRRT) during postoperative hospital stay.

Time-weighted average MAP (TWA-MAP) under 65 mmHg was calculated as the area under the curve (AUC) between 65 mmHg and the MAP measurements, divided by total CPB time. The AUC was computed using the composite trapezoidal rule (Fig. 1a).

**Fig. 1:**
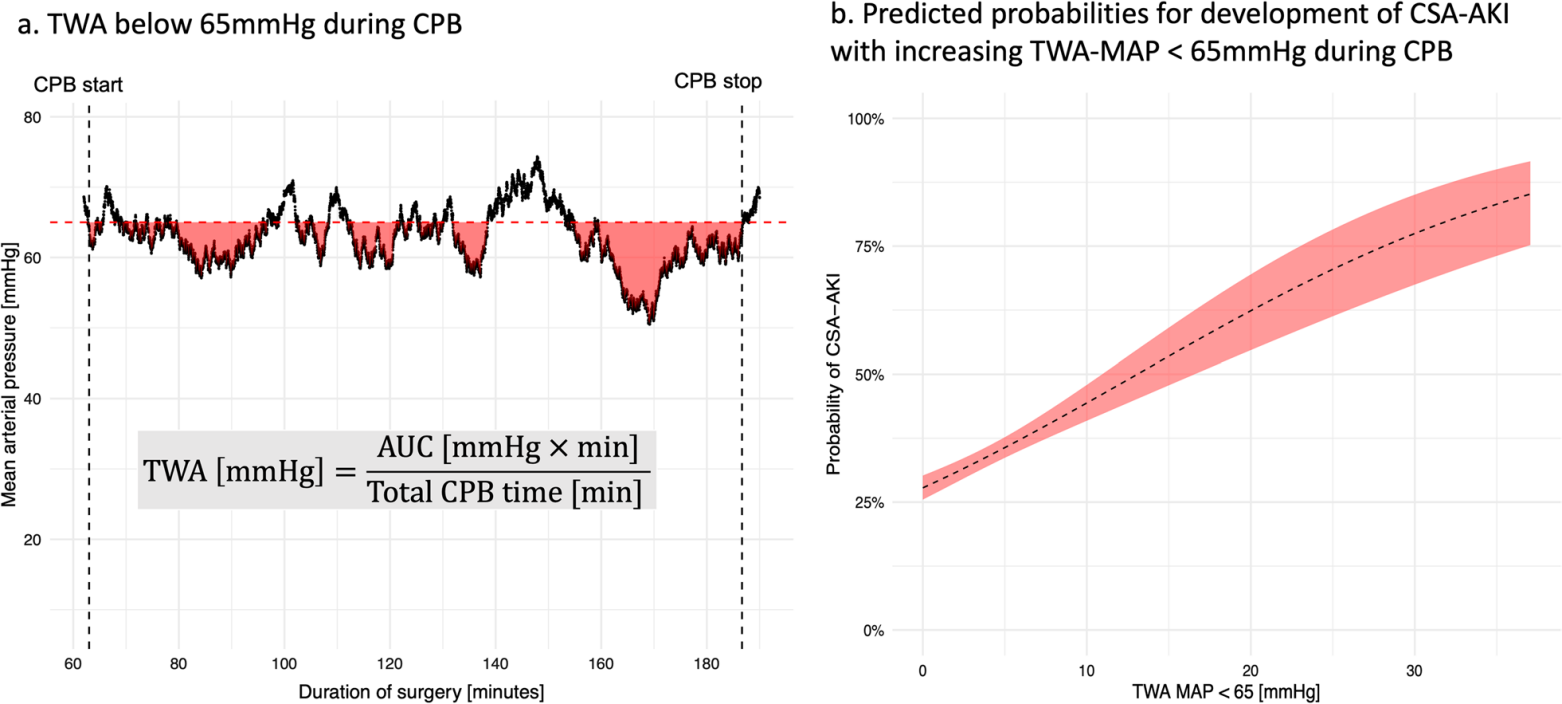
**a** An example of highly granular MAP data (with one second resolution) of a patient during CPB. The AUC between the threshold of 65 mmHg MAP and the respective MAP measurements is depicted as red shaded area. The AUC was calculated using the trapezoidal rule. Time-weighted average MAP under 65 mmHg was calculated as AUC divided by total CPB time. Start and stop of CPB are depicted as vertical black dashed lines, whereas the 65 mmHg MAP threshold is depicted as horizontal red dashed line. The x-axis shows the surgery times in minutes, and the y-axis shows the MAP in mmHg. **b** Predicted probabilities for the primary outcome of CSA-AKI derived from logistic regression models are shown. The 95%-CI is depicted as red shaded area. The data suggest a higher probability for CSA-AKI with increasing TWA-MAP under 65 mmHg during CPB. AUC—area under the curve; CSA-AKI—cardiac surgery-associated acute kidney injury; CI—confidence interval; CPB—cardiopulmonary bypass; MAP—mean arterial pressure; TWA—time-weighted average

Data are presented as numbers with percentages for categorical variables and medians with 25th percentile and 75th percentile for continuous variables.

To quantify the association between TWA-MAP under 65 mmHg and development of CSA-AKI, we fitted multivariable logistic regression models to estimate adjusted odds ratios (aORs) and 95% confidence intervals (CI). The models were adjusted for covariates, selected based on clinical plausibility. These are detailed in the 'Detailed Methods' section of our Additional file 1. Sensitivity analyses included a 75 mmHg cutoff for blood pressure and a subgroup examination separating patients with and without history of hypertension (see Additional file 1).

In total, 2352 patients were eligible for analysis. Thirty-four percent of patients (*n* = 802) developed CSA-AKI, and 10% of patients (*n* = 248) needed CRRT after surgery. The baseline demographics, intraoperative characteristics and results are summarized in Additional file 1: Table S1.

Median TWA-MAP under 65 mmHg was 3.0 (1.1–6.5) mmHg in patients who developed CSA-AKI, while it was 2.3 (0.7–4.7) mmHg in patients who did not (*p* < 0.001).

One mmHg increase in TWA-MAP under 65 mmHg yielded an aOR for development of CSA-AKI of 1.07 (95% CI 1.04–1.10, *p* < 0.001). The predicted probabilities for development of CSA-AKI with increasing TWA-MAP under 65 mmHg are shown in Fig. 1b.

In patients who received CRRT during their hospital stay after surgery, median TWA-MAP under 65 mmHg was 3.5 mmHg (1.1–7.2), whereas among patients who did not need CRRT, median TWA-MAP under 65 was 2.4 mmHg (0.8–5.0) (*p* < 0.001). This corresponded to an aOR of 1.05 (95% CI 1.01–1.10, *p* = 0.022) for receiving CRRT with every mmHg increase in TWA-MAP under 65 mmHg.

While prior studies are equivocal regarding the role of MAP during CPB in the development of CSA-AKI [2], our findings suggest that severity and duration of hypotension are strongly related to the occurrence of AKI and the need of CRRT after cardiac surgery.

To the best of our knowledge, this is the first study that evaluates TWA-MAP under 65 mmHg during the critical phase of CPB in association with AKI and renal replacement therapy in a sufficiently large and openly available dataset.

Remarkably, the association between TWA-MAP under 65 mmHg during CPB and CSA-AKI remained significant in both subgroups of patients, with and without history of hypertension.

Besides severity of hypotension, we observed a significant association between increased vasopressor dosage and CSA-AKI development in multivariable analysis, which may reflect the intricate relationship between renal perfusion, vasopressor exposition and consecutive CSA-AKI, meriting further research.

Our analysis is limited by its retrospective design and furthermore, despite thorough adjustment for confounding variables, it is important to note that all our analyses are exploratory, and association does not imply causation.

Nevertheless, our findings may provide a rationale to rethink blood pressure management during the phase of CPB.

Our results underscore the need for additional research and suggest that the inclusion of TWA-MAP < 65 in real-time monitoring deserves further investigation with the ultimate objective to develop standardized treatment protocols to mitigate the development of CSA-AKI.

### Supplementary Information

**Additional file 1: Table S1.** Baseline demographics, clinical characteristics and results. Data are reported as medians (with 25th–75th percentile in brackets) or as absolute counts (with percent in brackets). P-Values are derived from rank-sum tests. Odds ratios are derived from multivariable logistic regression models adjusted for covariates selected based on clinical plausibility: age, sex, weight, type of surgery, emergency surgery, ASA status, KDIGO—eGFR strata at admission, EuroSCORE II, diabetes mellitus, heart failure, hypertension, COPD, peripheral vascular disease, preoperative beta-blocker use, preoperative RAAS blockade, preoperative calcium antagonist use, total vasopressor-inotrope dose, fluid balance during surgery, TWA MAP under 65 mmHg during the post-CPB period, CPB time and units of pRBCs transfused intraoperatively. aORs—adjusted odds ratios; ASA—American Society of Anesthesiologists; AUC—area under the curve; CABG—coronary artery bypass grafting; CPB—cardiopulmonary bypass; CSA-AKI—cardiac surgery-associated acute kidney injury; CRRT—continuous renal replacement therapy; COPD—chronic obstructive pulmonary disease; eGFR—estimated glomerular filtration rate; KDIGO—Kidney Diseases: Improving Global Outcomes; MAP—mean arterial pressure; TWA—time-weighted average; pRBCs—packed red blood cells; RAAS—renin angiotensin aldosterone system. **Table S2.** Crude ORs for CSA-AKI from univariate logistic regression and adjusted ORs for CSA-AKI from multivariable logistic regression. aORs—adjusted odds ratios; ASA—American Society of Anesthesiologists; CABG—coronary artery bypass grafting; CI—confidence interval; COPD—chronic obstructive pulmonary disease; CPB—cardiopulmonary bypass; CSA-AKI—cardiac surgery-associated acute kidney injury; eGFR—estimated glomerular filtration rate; KDIGO—Kidney Diseases: Improving Global Outcomes; MAP—mean arterial pressure; min—minutes; pRBCs—packed red blood cells; RAAS—renin angiotensin aldosterone system; TWA—time-weighted average. **Table S3.** Time-weighted average mean arterial pressure under 75 mmHg and its effect on CSA-AKI and postoperative need of CRRT. We recalculated the TWA-MAP under 75 mmHg for both during and after the CPB period and then applied multivariable logistic regression models, adjusting for the same covariates as in our primary analysis. aORs—adjusted odds ratios; AUC—area under the curve; CSA-AKI—cardiac surgery-associated acute kidney injury; CI—confidence interval; CRRT—continuous renal replacement therapy; MAP—mean arterial pressure; TWA—time-weighted average. **Table S4**. Subgroup analysis separating patients with and without history of hypertension. The multivariable logistic regression models were adjusted for the same set of covariates as in the primary analysis except for prior hypertension. aORs—adjusted odds ratios; CSA-AKI—cardiac surgery-associated acute kidney injury; CI—confidence interval; MAP—mean arterial pressure; TWA—time-weighted average.

## Data Availability

The dataset used for this analysis is publicly available (https://physionet.org/content/inspire/1.2/). The code for data processing and analysis was written in R using RStudio (R Core Team (2022), R Foundation for Statistical Computing, Vienna, Austria, https://www.R-project.org/). Used packages include: stats, DescTools, tidyr, dplyr, ggplot2, car, glmtoolbox, gtsummary and caret. Main analysis code will be made available upon publication of the manuscript in the GitHub repository at https://github.com/schrnik/TWA_CPB.git

## Abbreviations

AKI: Acute kidney injury
aOR: Adjusted odds ratio
ASA: American Association of Anesthesiologists
CI: Confidence interval
CKD: Chronic kidney disease
CPB: Cardiopulmonary bypass
CRRT: Continuous renal replacement therapy
CSA-AKI: Cardiac surgery-associated acute kidney injury
eGFR: Estimated glomerular filtration rate according to the CKD-EPI-formula
KDIGO: Kidney Diseases: Improving Global Outcomes
MAP: Mean arterial pressure
pRBCs: Packed red blood cells
RAAS: Renin angiotensin aldosterone system
TWA: Time-weighted average
