# Supplementary material for: Mean arterial pressure during cardiopulmonary bypass: A modifiable risk factor for acute kidney injury in cardiac surgery patients?
Source: Crit Care. 2024 Mar 12;28:74. doi: 10.1186/s13054-024-04862-x (PMC10936033; doi:10.1186/s13054-024-04862-x)
Supplement: Supplementary file 1 — Additional file 1: Table S1. Baseline demographics, clinical characteristics and results. Data are reported as medians (with 25th–75th percentile in brackets) or as absolute counts (with percent in brackets). P-Values are derived from rank-sum tests. Odds ratios are derived from multivariable logistic regression models adjusted for covariates selected based on clinical plausibility: age, sex, weight, type of surgery, emergency surgery, ASA status, KDIGO—eGFR strata at admission, EuroSCORE II, diabetes mellitus, heart failure, hypertension, COPD, peripheral vascular disease, preoperative beta-blocker use, preoperative RAAS blockade, preoperative calcium antagonist use, total vasopressor-inotrope dose, fluid balance during surgery, TWA MAP under 65 mmHg during the post-CPB period, CPB time and units of pRBCs transfused intraoperatively. aORs—adjusted odds ratios; ASA—American Society of Anesthesiologists; AUC—area under the curve; CABG—coronary artery bypass grafting; CPB—cardiopulmonary bypass; CSA-AKI—cardiac surgery-associated acute kidney injury; CRRT—continuous renal replacement therapy; COPD—chronic obstructive pulmonary disease; eGFR—estimated glomerular filtration rate; KDIGO—Kidney Diseases: Improving Global Outcomes; MAP—mean arterial pressure; TWA—time-weighted average; pRBCs—packed red blood cells; RAAS—renin angiotensin aldosterone system. Table S2. Crude ORs for CSA-AKI from univariate logistic regression and adjusted ORs for CSA-AKI from multivariable logistic regression. aORs—adjusted odds ratios; ASA—American Society of Anesthesiologists; CABG—coronary artery bypass grafting; CI—confidence interval; COPD—chronic obstructive pulmonary disease; CPB—cardiopulmonary bypass; CSA-AKI—cardiac surgery-associated acute kidney injury; eGFR—estimated glomerular filtration rate; KDIGO—Kidney Diseases: Improving Global Outcomes; MAP—mean arterial pressure; min—minutes; pRBCs—packed red blood cells; RAAS—renin angiotensin aldosterone system; TWA—time-weighted avera [file 13054_2024_4862_MOESM1_ESM.docx]

Supplementary Table S1. Baseline demographics, clinical characteristics and results.

|  | **n=2352** |
| --- | --- |
| **Baseline Demographics** |  |
| Age | 65 (55 - 70) |
| Female | 913 (39%) |
| Weight [kg] | 62 (54 - 70) |
| Height [cm] | 163 (156 - 169) |
| **ASA** |  |
| I | 101 (4%) |
| II | 720 (31%) |
| III | 1359 (58%) |
| IV | 125 (5%) |
| V | 47 (2%) |
| **Comorbidities** |  |
| Diabetes mellitus | 598 (25%) |
| Heart failure | 339 (14%) |
| Hypertension | 420 (18%) |
| COPD | 129 (5%) |
| Peripheral vascular disease | 376 (16%) |
| EuroSCORE II | 1.50 (0.90 - 2.00) |
| **Preoperative medications** |  |
| Beta-blockers | 588 (25%) |
| RAAS-blockade | 494 (21%) |
| Calcium-antagonists | 447 (19%) |
| **KDIGO-eGFR strata at admission [ml/min/1,73m^2^]** |  |
| ≥ 90 | 1346 (57%) |
| 60 - 89 | 636 (27%) |
| 45 - 59 | 163 (7%) |
| 30 - 44 | 82 (3%) |
| 15 - 29 | 43 (2%) |
| < 15 | 82 (3%) |
| **Operative characteristics** |  |
| CABG | 1058 (45%) |
| Single valve | 435 (18%) |
| CABG + valve | 859 (37%) |
| Emergency surgery | 246 (10%) |
| Fluid balance [mL] | 1740 (950 – 2700) |
| TWA-MAP < 65mmHg during post-CPB period [mmHg] | 0.99 (0.18 – 2.92) |
| Total vasopressor inotrope dose [mg] | 0.84 (0.48 – 1.32) |
| pRBCs transfused intraoperatively [units] | 1 (1 – 2) |
| CPB time [min] | 195 (150 - 240) |
| Mean MAP during CPB [mmHg] | 67 (62 - 71) |

|  | **CSA-AKI (n=802)** | **No CSA-AKI (n=1550)** | **p-value** |
| --- | --- | --- | --- |
| TWA MAP < 65mmHg [mmHg] | 3.0 (1.1 - 6.5) | 2.3 (0.7 - 4.7) | **<0.001** |
| AUC [mmHg x min] | 668 (258 - 1520) | 386 (125 - 835) | **<0.001** |
|  | **CRRT (n=248)** | **No CRRT (n=2104)** |  |
| TWA MAP < 65 mmHg [mmHg] | 3.5 (1.1 - 7.2) | 2.4 (0.8 - 5.0) | **<0.001** |
| AUC [mmHg x min] | 752 (268 - 1861) | 440 (140 - 946) | **<0.001** |

|  | **aOR for development of CSA-AKI** | **95% CI** | **p-value** |
| --- | --- | --- | --- |
| TWA MAP < 65 mmHg per mmHg increase | 1.07 | 1.04 - 1.10 | **<0.001** |
|  | **aOR for need of CRRT** |  |  |
| TWA MAP < 65 mmHg per mmHg increase | 1.05 | 1.01 - 1.10 | **0.022** |

Detailed Methods:

All patients in the INSPIRE-dataset who underwent cardiopulmonary bypass (CPB) during cardiac surgery with availability of invasive mean arterial pressure data with at least five-minute resolution were included. Patients with preexisting dialysis-dependency were excluded.

Distribution of variables was assessed using rank-sum tests. We selected time weighted average mean arterial pressure (TWA-MAP) under 65mmHg as the measurement for hypotension because it reflects both the duration and severity of low blood pressure. To mitigate the influence of artifacts, the highest and lowest 1% of blood pressure measurements were excluded.

To quantify the association between the exposure of interest (TWA-MAP under 65mmHg during CPB) and the outcomes (occurrence of cardiac surgery associated acute kidney injury (CSA-AKI) according to Kidney Disease: Improving Global Outcomes (KDIGO) AKI-creatinine criteria (all stages) within 7 days of surgery and incidence of postoperative continuous renal replacement therapy) we fitted multivariable logistic regression models to derive adjusted Odds Ratios and 95%-Confidence Intervals.

The multivariable logistic regression models were adjusted for a predefined set of covariates, chosen based on clinical plausibility, which were subsequently refined based on suggestions from the peer review process. The covariates were incorporated into the models using the enter method. The covariates included: age, sex, weight, type of surgery, emergency surgery, ASA- status, KDIGO – eGFR-strata at admission, EuroSCORE II, diabetes mellitus, heart failure, hypertension, COPD, peripheral vascular disease, preoperative beta-blocker use, preoperative RAAS-blockade, preoperative calcium antagonist use, total vasopressor-inotrope dose, fluid balance during surgery, TWA-MAP under 65mmHg during the post-CPB period, CPB-time and units pRBCs transfused intraoperatively.

In a first step we performed univariate analysis to assess if the covariates chosen based on clinical relevance show univariate association with the outcomes.

The linearity assumption of the effects of the predictor variables on the log odds of the outcome variables was assessed using restricted cubic splines. Variance inflation factor (VIF) was used to assess multicollinearity between the predictor variables. The Hosmer–Lemeshow goodness-of-fit test was applied to assess the model fit. The results of the logistic regression analyses are shown in Supplementary Table S2, including crude ORs from univariate analyses and adjusted ORs from the multivariable model. All tests were two-sided, with 0.05 as the level of significance.

Total vasopressor-inotrope dose was calculated using the following formula: *Total vasopressor-inotrope dose = [norepinephrine (μg/min) × min] + [epinephrine (μg/min) × min] + [(phenylephrine (μg/min) × min) ÷ 10] + [vasopressin (U/h) × 8.33 × min]* as used by de la Hoz et al. (1)

The TWA-MAP under 65mmHg during the post-CPB period was calculated using the same methodology as for the TWA-MAP under 65mmHg during the CPB-period. However, for the post-CPB period, the area under the curve (AUC) between 65 mmHg and the actual MAP measurements was divided by the duration from the end of CPB to the end of the surgery.

Supplementary Table S2. Crude ORs for CSA-AKI from univariate logistic regression and adjusted ORs for CSA-AKI from multivariable logistic regression

| **Variable** | **Crude OR (95% CI)** | **p-value** | **aOR (95% CI)** | **p-value** |
| --- | --- | --- | --- | --- |
| TWA MAP < 65 mmHg during CPB per mmHg increase | 1.08 (1.06 - 1.10) | **<0.001** | 1.07 (1.04 - 1.10) | **<0.001** |
| Age (per 5 years increase) | 1.03 (1.02 – 1.04) | **<0.001** | 1.03 (1.02 – 1.04) | **<0.001** |
| Sex (male vs. female) | 0.89 (0.75 – 1.07) | 0.234 | 1.12 (0.84 - 1.49) | 0.543 |
| Weight (per 10kg increase) | 0.98 (0.97 – 0.99) | **<0.001** | 0.98 (0.97 – 1.02) | 0.091 |
| ***Type of surgery*** |  |  |  |  |
| Single valve vs. CABG | 0.92 (0.78 – 1.22) | 0.616 | 0.95 (0.68 – 1.32) | 0.834 |
| CABG+valve vs. CABG | 0.90 (0.85 – 1.15) | 0.382 | 0.89 (0.69 – 1.15) | 0.432 |
| Emergency surgery (yes vs. no) | 2.18 (1.67 – 2.85) | **<0.001** | 1.02 (0.67 – 1.53) | 0.914 |
| ***ASA*** |  |  |  |  |
| II vs. I | 0.96 (0.91 – 1.14) | 0.127 | 0.85 (0.46 – 1.61) | 0.634 |
| III vs. I | 1.02 (1.01 – 1.28) | **0.041** | 0.86 (0.57 – 1.85) | 0.734 |
| IV vs. I | 1.37 (1.03 – 3.43) | **0.045** | 1.02 (0.47 – 3.64) | 0.812 |
| V vs. I | 1.65 (1.21 – 4.32) | **0.048** | 0.97 (0.88 – 4.56) | 0.926 |
| ***KDIGO-eGFR strata at admission [ml/min/1,73m^2^]*** |  |  |  |  |
| 60 – 89 vs. ≥ 90 | 1.18 (0.89 – 1.21) | 0.478 | 1.22 (0.46 – 1.24) | 0.673 |
| 45 – 59 vs. ≥ 90 | 1.20 (0.75 – 3.42) | 0.541 | 1.44 (0.42 – 1.53) | 0.794 |
| 30 – 44 vs. ≥ 90 | 1.55 (1.23 – 4.58) | **<0.001** | 1.37 (1.01 – 4.87) | **0.045** |
| 15 – 29 vs. ≥ 90 | 1.83 (1.45 – 5.02) | **<0.001** | 1.77 (1.39 – 5.14) | **0.037** |
| < 15 vs. ≥ 90 | 3.15 (2.13 – 5.53) | **<0.001** | 2.87 (1.91 – 5.63) | **0.023** |
| ***Comorbidities*** |  |  |  |  |
| EuroSCORE II (per percent increase) | 1.38 (1.17 – 1.62) | **<0.001** | 1.21 (1.02 – 1.67) | **0.043** |
| Diabetes mellitus | 1.39 (1.14 – 1.68) | **<0.001** | 1.38 (1.04 – 1.84) | **0.026** |
| Heart failure | 2.98 (2.36 – 3.78) | **<0.001** | 2.11 (1.51 – 2.95) | **<0.001** |
| Hypertension | 1.64 (1.32 – 2.03) | **<0.001** | 1.65 (1.22 – 2.23) | **0.001** |
| COPD | 2.01 (1.41 – 2.88) | **<0.001** | 1.54 (0.94 – 2.51) | 0.082 |
| Peripheral vascular disease | 1.42 (1.31 – 1.56) | **<0.001** | 1.32 (1.12 – 1.57) | **0.019** |
| ***Preoperative medications*** |  |  |  |  |
| Preoperative beta-blocker use | 1.22 (0.99 – 1.47) | 0.059 | 0.97 (0.74 – 1.26) | 0.834 |
| Preoperative RAAS-blockade | 0.95 (0.76 – 1.17) | 0.637 | 1.04 (0.78 – 1.38) | 0.818 |
| Preoperative calcium antagonist use | 1.12 (0.90 – 1.39) | 0.293 | 1.19 (0.89 – 1.60) | 0.276 |
| ***Operative characteristics*** |  |  |  |  |
| Total vasopressor-inotrope dose (per mg increase) | 1.65 (1.32 – 1.82) | **<0.001** | 1.55 (1.26 – 1.91) | **<0.001** |
| Fluid balance during surgery (per 250mL increase) | 1.00 (0.99 – 1.01) | 0.067 | 1.01 (0.97 – 1.23) | 0.245 |
| TWA MAP < 65 mmHg during the post-CPB period per mmHg increase | 1.04 (1.03 – 1.09) | **<0.001** | 1.02 (1.01 – 1.07) | **<0.001** |
| CPB-time (per 10 min. increase) | 1.01 (1.00 – 1.02) | **<0.001** | 1.01 (1.00 – 1.01) | **<0.001** |
| pRBCs transfused intraoperatively |  |  |  |  |
| 1 vs. 0 | 1.25 (0.97 – 1.39) | 0.318 | 1.21 (0.93 – 1.34) | 0.412 |
| 2 vs. 0 | 1.47 (1.21 – 1.65) | **0.001** | 1.42 (0.85 – 1.47) | 0.653 |
| 3 vs. 0 | 1.81 (1.11 – 2.01) | **<0.001** | 1.77 (1.03 – 2.45) | **0.003** |
| ≥ 4 vs. 0 | 3.05 (2.78 – 5.27) | **<0.001** | 2.89 (2.48 – 6.73) | **<0.001** |

Sensitivity analyses

To evaluate the robustness of our results under varying conditions, we conducted sensitivity analyses. Initially, we considered a higher hypotension threshold, setting the cut-off at 75 mmHg. For this, we recalculated the TWA-MAP under 75 mmHg for both during and after the CPB period. We then applied multivariable logistic regression models, adjusting for the same covariates as in our primary analysis. The results are displayed in Supplementary Table S3.

Additionally, we explored the influence of pre-existing hypertension on our findings by conducting a subgroup analysis, separating patients with and without prior history of hypertension. The multivariable logistic regression models were adjusted for the same set of covariates as in the primary analysis except for prior hypertension. The results are shown in Supplementary Table S4.

Supplementary Table S3 – Time weighted average mean arterial pressure under 75mmHg and its effect on CSA-AKI and postoperative need of CRRT

|  | **CSA-AKI (n=802)** | **No CSA-AKI (n=1550)** | **p-value** |
| --- | --- | --- | --- |
| TWA MAP < 75 mmHg [mmHg] | 10.2 (6.3 – 15.0) | 8.9 (5.6 – 12.6) | **<0.001** |
| AUC [mmHg x min] | 2158 (1305 - 3379) | 1559 (912 - 2431) | **<0.001** |
|  | **CRRT (n=248)** | **No CRRT (n=2104)** |  |
| TWA MAP < 75 mmHg [mmHg] | 10.8 (6.6 – 15.1) | 9.2 (5.8 – 13.1) | **<0.001** |
| AUC [mmHg x min] | 2460 (1464- 4205) | 1658 (988 - 2612) | **<0.001** |

|  | **aOR for development of CSA-AKI** | **95% CI** | **p-value** |
| --- | --- | --- | --- |
| TWA MAP < 75 mmHg per mmHg increase | 1.05 | 1.03 - 1.07 | **<0.001** |
|  | **aOR for need of CRRT** |  |  |
| TWA MAP < 75 mmHg per mmHg increase | 1.03 | 1.01 - 1.07 | **0.041** |

Supplementary Table S4 – Subgroup analysis separating patients with and without history of hypertension

| **Patients with history of hypertension (n=420)** | | | |
| --- | --- | --- | --- |
|  | **aOR for development of CSA-AKI** | **95% CI** | **p-value** |
| TWA MAP < 65 mmHg per mmHg increase | 1.11 | 1.04 - 1.20 | **0.004** |
| **Patients without history of hypertension (n=1932)** | | | |
| TWA MAP < 65 mmHg per mmHg increase | 1.06 | 1.02 - 1.10 | **0.004** |

References

1. de la Hoz MA, Rangasamy V, Bastos AB, Xu X, Novack V, Saugel B, et al. Intraoperative Hypotension and Acute Kidney Injury, Stroke, and Mortality during and outside Cardiopulmonary Bypass: A Retrospective Observational Cohort Study. Anesthesiology. 2022 Jun 1;136(6):927–39.
